# Comparative histological study of hepatic architecture in the three orders amphibian livers

**DOI:** 10.1186/1476-5926-11-2

**Published:** 2012-08-20

**Authors:** Hideo Akiyoshi, Asuka M Inoue

**Affiliations:** 1 Department of Biological Science, Faculty of Life and Environmental Science, Shimane University, Matsue, Japan

**Keywords:** Liver, Amphibian, Hematopoietic tissue, Phylogeny, Evolution

## Abstract

**Background:**

This report presents a detailed description of hepatic architecture in 46 amphibian livers by light microscopy, and extensively discusses the phylogenetic viewpoint.

**Results:**

The 46 amphibian livers showed a variety of histological features, but anurans were the same as in mammalian livers. The hepatocyte-sinusoidal structures of the amphibian livers were classified into three different types: (I) several-cell-thick plate type, (II) two-cell-thick plate type, and (III) one-cell-thick plate type, depending on the percentage extension of sinusoidal areas per unit area, measured by morphometry. Hematopoietic tissue structures were observed in the connective tissue of both the perihepatic subcapsular regions and portal triads in the order Caudata and Gymnophiona, but were not observed in the order Anura (except for the genus *Bombina* and *Xenopus*). As phylogenetic relationships are branched from urodeles to anurans, the parenchyma arrangement progressed from the combined several- and two-cell-thick plate type to one-cell-thick plate type as seen in the mammalian liver type. In contrast, hematopoietic tissue structures were exactly the opposite and did not involve anurans.

**Conclusions:**

This study is the first to investigate amphibian livers phylogenically, and their architectural differences are shown in the route of hepatic ontogenesis. In this process, parenchymal arrangement formation is acquired phylogenically. The occurrence of hematopoietic cells may be related with the development of the systemic immune system in the spleen and bone marrow.

## Introduction

The liver plays an indispensable part in many processes in the body, particularly those concerned with its metabolism (e.g., protein synthesis, storage metabolites, bile secretion and detoxification) that shoulder a central role into maintaining life, and with certain digestive processes. It is the organ in which nutrients absorbed in the digestive tract are processed and stored for use by other parts of the body.

The usual concept of structural and functional unit of the liver is the acinus, containing both the hepatic lobule and portal triad. The hepatic lobule is formed hepatocyte-sinusoidal structures in which consist of both hepatocytes and sinusoids. The sinusoids are capillary networks and are localized in the space between hepatic plates in which hepatocytes are arranged [1]. In mammals, hepatic plates line simple-layered hepatocytes, so-called one-cell-thick plates or with a cord-like form [2]. In teleosts, hepatic plates line the multi-layered hepatocytes, so-called two- or several-cell-thick plates and/or solid or tubular types [2,3].

The portal triads are located in the portal spaces between the hepatic lobules and contain branches of the portal vein and hepatic artery, bile duct and lymph vessels which are surrounded by connective tissue. In amphibians, the liver of the newt possesses immunologic capabilities due to the presence of lymphocytes in both the connective tissue region in the portal triad and the perihepatic subcapsular region [4,5]. It is the site of formation of lymphocytes and of the eosinophil leukocytes. In contrast, mice and humans, except the fetal liver, hematopoietic tissue structures are not possessed in these regions. The fetal liver has the initial site of fetal hematopoiesis [6,7] and B cell development in mammals [8].

In amphibian livers, a number of morphological studies have been performed. The recent aims of the amphibian liver have been as follows: (1) animal diversity and evolution (e.g., phylogeny, ontogeny, and taxonomy), (2) immunological mechanism (e.g., lymphoid system and pigment system), and (3) pollution (e.g., endocrine disruptors). Evolutionary or phylogenetic relationships among the families of living amphibians are basic to an interpretation of their biography and to constructing a meaningful classification. The current zoological viewpoints have been focused and investigated in the themes of biodiversity or evolution, but there has been little phylogenic research into any vertebrates in liver evolution [9-16]. On the other hand, the interaction of hepatocyte-sinusoidal structures with phylogeny in several vertebrate species has been elucidated [2,3]; however, there is no study among each order in amphibians.

Amphibians can be grouped into three orders: Gymnophiona, Caudata and Anura [17-19]. Gymnophiona are elongate, legless, wormlike animals that live primarily in tropical areas. Caudata include newts and salamanders, and newts are aquatic members of the Salamandridae family. Anurans include tailless toads and frogs. The adults of most species are terrestrial, although the genus *Xenopus* is an aquatic member of the Pipidae family [20].

The origin and divergence of the three living orders of amphibians (Gymnophiona, Caudata, Anura) and their main lineages are one of the most hotly debated topics in vertebrate evolution [19]. A phylogenic study of amphibian livers may be valid as an optimal model for liver ontogenesis in vertebrates. To demonstrate the correlation between liver structures and phylogenic status, we observed 46 amphibian livers by light microscope, and subjected the data to phylogenic analyses. We focused on the architecture of hepatocyte-sinusoidal structures and hematopoietic tissue structures.

## Methods

The present study was approved by the animal ethics committee of Shimane University, and carried out in strict accordance with the guidelines for the care and use of research animals set by the committee.

### Sample collection

For this comparative morphological study, the livers of 46 different amphibian species were used. Using hand nets, we collected 21 species from ponds and streams in Shimane Prefecture, 8 species in Iriomote Ishigaki and Miyako Islands in Okinawa Prefecture, 4 species in Amami-oosihma Islands in Kagoshima Prefecture, 2 species in Hokkaidou, 2 species in Aomori Prefecture, 1 species in Oita Prefecture, 1 species in Miyazaki Prefecture, 1 species in Nagasaki Prefecture, 1 species in Gifu Prefecture, and 1 species in Hyogo Prefecture Cayenne caecilians (*Typhlonectes* sp), Oriental fire-bellied toads (*Bombina orientalis*) and African clawed frogs (*Xenopus laevis* and *Xenopus tropicalis*) were reared in the Biological Fresh Water Laboratory, Shimane University. In order to eliminate the influence of seasonal changes or growth, all specimens were both male and female in the adult stage, anurans were caught from April to October, and urodeles were caught from December to March in each locality from 2005 to 2010. Three to five specimens were sampled, respectively, except for Japanese giant salamander (*Andrias japonicus*) of which one sample was transported to our laboratory by accident. Animals were anesthetized by immersion in an ice water bath in 2 ml/L aqueous ethylene glycol monophenyl ether (Merck). After deep anesthetization, liver was taken from the animal. The phylogenetic relationships of Amphibian Class, comprising three orders of amphibian: 13 urodeles, 1 caecilian, and 32 anurans species, is shown in Table 1.

**Table 1:** Summary of the phylogenetic relationships in Amphibian Class

**Order**	**Suborder**	**Family**	**Species number**
Gymnophiona		Typhlonectidae	1
Caudata	Cryptobranchoidea	Hynobiidae	10
	Salamandroidea	Cryptobranchidae	1
		Salamandriae	2
Anura	Archaeobatrachia	Discoglossidae	1
	Aglossa	Pipidae	2
	Neobatrachia	Bufonidae	4
		Hylidae	1
		Ranidae	17
		Rhacophoridae	6
		Microhylidae	1

Table includes 13 urodeles, 1 caecilian, and 32 anuran species (Class: Amphibia; Subclass: Lissamphibia).

### Histology

The livers were perfusion-fixed via the heart with 4% paraformaldehyde buffered at pH 7.4 with 0.1 M phosphate for 15 min, cut into small pieces, and immersed in the same solution for 3 days at 4°C. The specimens were rinsed, dehydrated and embedded in paraffin. Serial 4 μm sections were obtained, and some were stained with both hematoxylin and eosin, and others were stained with Azan for morphometric analysis.

### Morphometry

Morphometry is the evaluation of forms and in histology describes measurements made from two-dimensional sections. Liver sections stained with Azan for hepatic cells were subjected to morphometric analysis. We used a computerized image analysis system comprised of a photomicroscope (Olympus BX51) and digital camera (Olympus DP70) and the Lumina Vision software (Mitani Corporation, Tokyo, Japan). Lumina Vision is a Microsoft Windows XP application of semi-automated quantitative analysis of fixed histological sections. The software performs automatic measurement of areas defined using an interactive threshold editing functions. The latter results in colored overlay that marks which pixels in the image are to be measured. In the current study, the percentage extension of the sinusoidal areas was quantified in two different zones: periportal zone (PZ) in around the portal triad and pericentral zone (CZ) in around the central vein in hepatic lobules. At least three areas, 6 random fields in each section were captured on digitalized images at a final magnification of 200X. The analysis in each species was made from 18 randomly chosen zones in three specimens.

## Results

The results of hematoxylin and eosin staining for hepatocyte-sinusoidal structures and hematopoietic tissue structures in the livers of 46 amphibians are summarized in Table 2 and 3. The 46 amphibian livers varied in their microscopic images, but anurans had the same image as is seen in mammalian livers.

**Table 2:** Summary of the expression levels of hepatocyte-sinusoidal structures and hematopoietic tissue structures in livers of Urodela and Gymnophiona species

**Order**	**Order**	**Species**	**HSS**	**Hematopoietic tissue structures**		
	**Family**		**PZ | CZ**	**PSR**	**PTR**	**IHLN**
Urodela	Cryptobranchoidea					
	Hynobiidae	*Hynobius nebulosus*	2 | 1	+	+	+
		*Hynobius dunni*	2 | 1	+	+	+
		*Hynobius naevius*	2 | 1	+	+	+
		*Hynobius okiensis*	3 | 2	+	+	+
		*Hynobius stejnegeri*	3 | 2	+	+	+
		*Hynobius kimurae*	3 | 2	+	+	+
		*Hynobius nigrescens*	3 | 2	+	+	+
		*Hynobius lichenatus*	3 | 2	+	+	+
		*Hynobius retardatus*	3 | 3	+	+	+
		*Onychodactylus japonicus*	3 | 3	+	+	+
	Cryptobranchoidea					
	Cryptobranchidae	*Andrias japonicus*	3 | 2	+	+	+
	Salamandroidea					
	Salamandridae	*Cynops ensicauda*	3 | 2	+	+	+
		*Cynops pyrrhogaster*	3 | 2	+	+	+
Gymnophiona	Typhlonectidae	*Typhlonectes* sp.	3 | 3	+	+	+

Hepatocyte-sinusoidal structure (HSS): (1) several-cell-thick plate type, (2) two-cell-thick plate type, (3): one-cell-thick plate type. Hematopoietic tissue structures: (−): do not exist, (+): exist. CZ – pericentral zone; IHLN – Inter-hepatic lobular nodule; PZ – periportal zone; PSR – Perihepatic subcapsular region; Portal triad region – PTR.

**Table 3:** Summary of the expression levels of hepatocyte-sinusoidal structures and hematopoietic tissue structures in livers of Anura species

**Order**	**Order**	**Species**	**HSS**	**Hematopoietic tissue structures**		
	**Family**		**PZ | CZ**	**PSR**	**PTR**	**IHLN**
Anura	Archaeobatrachia					
	Discoglossidae	*Bombina orientalis*	3 | 3	+	+	+
	Aglossa					
	Pipidae	*Xenopus laevis*	3 | 2	+	+	+
		*Xenopus tropicaris*	3 | 2	+	+	+
	Neobatrachia					
	Bufonidae	*Bufo gargarizans miyakonis*	3 | 3	-	-	+
		*Bufo japonicus japonicus*	3 | 3	-	-	-
		*Bufo japonicus formosus*	3 | 3	-	-	-
		*Bufo marinus*	3 | 3	-	-	-
	Hylidae					
		*Hyla japonica*	3 | 3	-	-	-
	Ranidae					
		*Rana japonica*	3 | 3	-	-	+
		*Rana tsushimensis*	3 | 3	-	-	-
		*Rana tagoi tagoi*	3 | 3	-	-	+
		*Rana tagoi okiensis*	3 | 3	-	-	-
		*Rana sakuraii*	3 | 3	-	-	-
		*Rana pirica*	3 | 3	-	-	-
		*Rana ornativentris*	3 | 3	-	-	+
		*Rana nigromaculata*	3 | 3	-	-	+
		*Rana porosa brevipoda*	3 | 3	-	-	+
		*Rana rugosa*	3 | 3	-	-	+
		*Rana catesbeiana*	3 | 3	-	-	-
		*Rana limnocharis*	3 | 3	-	-	+
		*Rana sp.*	3 | 3	-	-	+
		*Rana supranarina*	3 | 3	-	-	-
		*Rana utsunomiyaorum*	3 | 3	-	-	-
		*Rana psaltes*	3 | 2	-	-	+
		*Rana subaspera*	3 | 3	-	-	-
	Rhacophoridae					
		*Buergeria buergeri*	3 | 3	-	-	+
		*Buergeria japonica*	3 | 3	-	-	+
		*Rhacophorus arboreus*	3 | 3	-	-	+
		*Rhacophorus viridis amamiensis*	3 | 3	-	-	-
		*Rhacophorus schlegelii*	3 | 3	-	-	
		*Rhacophorus owstoni*	3 | 3	-	-	+
	Microhylidae					
		*Microhyla ornata*	3 | 2	-	-	+

Hepatocyte-sinusoidal structure (HSS): (1) several-cell-thick plate type, (2) two-cell-thick plate type, (3): one-cell-thick plate type. Hematopoietic tissue structures: (−): do not exist, (+): exist. CZ – pericentral zone; IHLN – Inter-hepatic lobular nodule; PZ – periportal zone; PSR – Perihepatic subcapsular region; Portal triad region – PTR.

**Figure 1:**
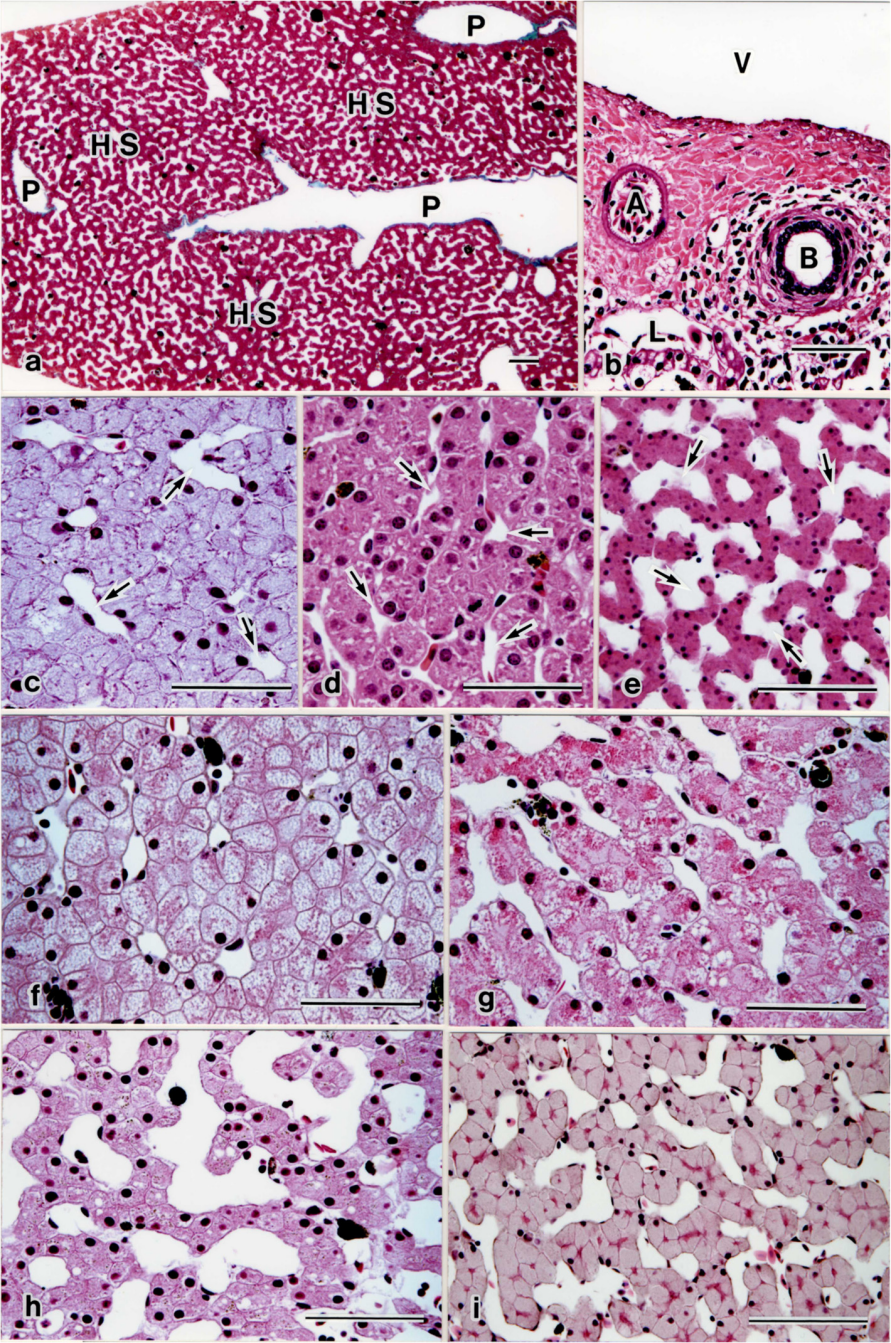
**Light micrographs of the liver.** Low magnification light micrographs of hepatic lobule in livers. (**a**) A portal triad (P) is seen in the hepatic lobule, and consists of both hepatocytes and sinusoidal blood capillary networks, in which hepatocyte-sinusoidal structures (HS) are formed. Montane brown frog (*Rana ornativentris*). (**b**) High magnification light micrograph of portal triad. A portal space with its characteristic small hepatic artery (A) portal vein (V), lymph vessel (L), and bile duct (B) is surrounded by connective tissue. Japanese giant salamanders (*Andrias japonicus*). High magnification light micrographs of hepatocyte-sinusoidal structures in livers. (**c**) Several-cell-thick plate type. The hepatocyte lining is multi-layered. Hepatic sinusoids (arrows) are narrow and short tortuous capillaries. Hepatocytes are rounded, and have a small rounded nucleus. Clouded salamander (*Hyobius nebulosus*). (**d**) Two-cell-thick plate type. T he hepatocyte lining is double-layered. Sinusoidal capillaries (arrows) are narrow and irregularly shaped sinusoids appearing throughout the interstices between the hepatic plates. Hepatocytes are polyhedral or rounded and have a rounded nucleus. Amber-colored salamander (*Hynobius stejnegeri*). (**e**) One-cell-thick plate type. The hepatocyte lining is simple-layered. Hepatic sinusoids (arrows) are enlarged with straight capillaries. Hepatocytes are polyhedral and have a rounded nucleus. Montane brown frog (*Rana ornativentris*). (**f**) Genus *Hynobius* are of the combined several- and two-cell-thick plate type. Hepatocytes are rounded and have a large nucleus. Spotted salamander (*Hynobius naevius*). (**g**) Another genus of the *Hynobius* group is of the combined one- and two-cell-thick plate type. Hepatocytes are square and have a large nucleus. Hida salamander (*Hynobius kimurae*). (**h**) In the order Gymnophiona, the parenchyma arrangement is one-cell-thick plate type. Sinusoidal capillaries are enlarged. Hepatocytes are square, and have a large rounded large nucleus. Cayenne caecilian (*Typhlonectes* sp.). (**i**) In the order Anura, the parenchyma arrangement is the one-cell-thick plate type. Sinusoidal capillaries are enlarged. Hepatocytes are square and polyhedral and have a small rounded nucleus. Schlegel’s green frog (*Rhacophorus schlegelii*). Scale bars = 100 μm.

All amphibian livers were observed in the hepatic lobules (Figure 1a), known as structural units, demarcated by connective tissue septa shown as the portal triad (portal tract), which contain bile ducts, portal and arterial vessels. These vessels and ducts are surrounded by connective tissue (Figure 1b). The hepatic lobules consisted of both hepatocytes and sinusoidal blood capillary networks, in which hepatocyte-sinusoidal structures are formed (Figure 1a). Sinusoids are localized in the space between hepatic plates in which hepatocytes are arranged.

### Hepatocyte-sinusoidal structures

Following cardiac perfusion fixation, hepatic sinusoids were cleared of blood cells and the definition of hepatocyte-sinusoidal structures was enhanced. Depending on the percentage of hepatic sinusoids per unit area, measured by morphometry, hepatocyte-sinusoidal structures of amphibian livers were divided into three classes as follows: class I (percentage 5 to < 15), class II (percentage 15 to < 25) and class III (percentage ≥ 25).

Histologically, in hepatocyte-sinusoidal structures, class I showed the several-cell-thick plate type, the major part of the hepatocyte lining was multi-layered. The hepatic sinusoids were narrow and short tortuous capillaries. The hepatocytes were rounded and had a rounded large nucleus (Figure 1c). In class II, hepatocyte-sinusoidal structures were observed in the two-cell-thick plate type, the majority of the hepatocyte lining was double-layered. The sinusoidal capillaries were narrow with irregularly shaped sinusoids appearing throughout the interstice between the hepatic plates. Three to four hepatocytes surrounded a sinusoidal capillary. The hepatocytes were polyhedral or rounded, and had a large rounded nucleus (Figure 1d). Class III showed the one-cell-thick plate type, the majority of the hepatocyte lining was simple-layered. The hepatic sinusoids were enlarged with straight capillaries connecting through the perilobular to the centrolobular vessels. The hepatocytes were polyhedral, with a small rounded nucleus (Figure 1e).

In the order Caudata, the hepatocytes were rounded, and had a large rounded nucleus. The sinusoidal capillaries were narrow with short tortuous capillaries. The parenchyma arrangements of some genus *Hynobius* (*nebulosus*, *dunni*, and *naevius*) were of the combined several- and two-cell-thick plate types (Figure 1f), but other genus *Hynobius* groups, genus *Andrias* and the Salamandridae family were of the combined one- and two-cell-thick plate type (Figure 1g). A few urodeles, (*Hynobius retardatus*, *Onnychodactylus japonicus*, and *Cynops pyrrhogaster*), are shown as the one-cell-thick plate type. In the order Gymnophiona, the hepatocytes were square, and had a large rounded nucleus. The sinusoidal capillaries were enlarged. The parenchyma arrangement was the one-cell-thick plate type (Figure 1h). In the order Anura, the hepatocytes were square and polyhedral, and had a small rounded nucleus. The sinusoidal capillaries were enlarged, and the parenchyma arrangement was the one-cell-thick plate type (Figure 1i).

### Hematopoietic tissue structures

**Figure 2:**
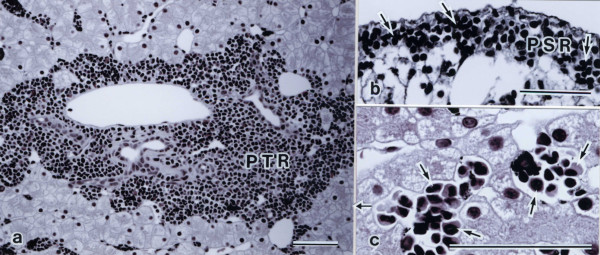
**High magnification light micrographs of hematopoietic tissue structures in the liver.** (**a**) Portal triad region (PTR). Numerous hematopoietic cells are seen in the connective tissue of the portal space. Spotted salamanders (*Hynobius naevius*). (**b**) Perihepatic subcapsular region (PSR). PSR is usually two to six cell layers thick, almost completely enveloping the hepatic parenchyma, with the visceral peritoneum adjoining (arrows). This tissue contains neutrophils (arrows) and eosinophils. African clawed frog (*Xenopus laevis*). (**c**) Inter-hepatic lobular nodule. Numerous hematopoietic cells (arrows) are seen in the sinusoidal capillaries of the hepatic lobule. Sakishima rice frog (*Rana* sp.). Scale bars = 100 μm.

Hematopoietic tissue structures were observed in the three regions: (a) portal triad region (PTR), (b) perihepatic subcapsular region (PSR), and (c) inter-hepatic lobular nodule (Figures 2a-c). In PTR, numerous hematopoietic cells were observed in the connective tissue (Figure 2a). The PSR, usually two to six cell layers thick, almost completely enveloped the hepatic parenchyma, with occasional sites where hepatic parenchymal cells and visceral peritoneum adjoined. This tissue contained neutrophils and eoshinophils (Figure 2b). In the hepatic lobule, hematopoietic nodules were observed in the sinusoidal capillaries with involvement in the Kupffer cells (Figure 2c).

In the order Caudata, the liver consisted of several incompletely separated lobes of parenchymal tissue, each of which was covered by a PSR of hematopoietic tissue. Hematopoietic tissue was also shown in both the portal triads, and was also observed in the inter-hepatic nodule. In the order Gymnophiona, the liver also consisted of several incompletely separated lobes of parenchymal tissue, each of which was covered by a PSR of hematopoietic tissue. Hematopoietic tissue was also shown in both the portal triads and inter-hepatic nodules. In the order Anura, the liver of most anurans was not observed in the hematopoietic tissue structure, but the liver of the genus *Bombina* and *Xenopus* was observed in the PSR. Hematopoietic nodules were observed in the hepatic lobule in most anuran amphibians.

## Discussion

This study is the first to investigate amphibian livers phylogenically. We aimed to identify the interrelation of hepatocytes, sinusoids, and hematopoietic tissue, and make a comparison with phylogenic development.

### Circulatory capillaries arrangement in the liver

All ingested materials are absorbed via the intestines, and reach the liver through the portal vein. Blood flows from the portal veins at the portal triads through the sinusoid and between the hepatic plates to the central vein. The hepatocyte-sinusoidal structure is physiologically important, not only because hepatocytes take up large molecules (e.g., amino acids, glucose, and vitamins) from the sinusoid, but also because a large number of macromolecules (e.g., lipoproteins and albumin) are secreted into the sinusoid [1]. In mammalian livers, hepatocytes are closely contacted with sinusoidal capillaries that form a dense network [2]. In teleosts, hepatocyte-sinusoidal structures are shown as a rough network [1-3,13].

This study has shown that the hepatocyte-sinusoidal structures of amphibian livers can be classified into three different types: (I) several-cell-thick plate type, (II) two-cell-thick plate type, and (III) one-cell-thick plate type. This classification is based on the investigation of Elias and Bengelsdorf in several vertebrate animals [2]. Previous studies described that some fish had a similar structure to normal humans, while others were modified in a more primitive form [3,21]. Our study of 46 species showed that the primitive form was a combination of several-cell-thick plate and two-cell-thick plate types in the genus Hynobius. The traditional form was the combined two- and one-cell-thick plate type, and was observed in another genus, the *Hynobius* group, genus *Andrias* and the Salamandridae family. The mammalian form was the one-cell-thick plate type, and was observed in the order Anura, order Gymnophiona and part of the order Caudata. It is well known that the phylogenetic relationships in amphibians is clearly categorized (Table 1). Anura is the sister group of Caudata to the exclusion of Gymnophiona [18].

In this study, we revealed that anuran livers had structures identical to the mammalian arrangement, which possess higher metabolic functions. In contrast, urodeles livers had sinusoids of a primitive form, which were narrow with an undeveloped network, identical to teleosts. As phylogenetic relationships are branched from urodeles to anurans, the parenchyma arrangement progressed from the combined several- or two-cell-thick plate to the one-cell-thick plate type, and the hepatocytes changed from round to square and polyhedral cells. We speculated that these structural changes reflect the route of hepatic ontogenesis, and are essential for the acquisition of higher hepatic function.

### Hematopoiesis in the liver

The liver develops as a hematopoietic organ at the fetal stage in the mammalian liver, prior to bone marrow development [8]. In amphibians, the liver is an immunocompetent organ, and hepatic hematopoiesis is initiated in urodele sites. It is well known that the thymus, spleen and liver are the three primary sites of hematopoiesis in the adult newt [5,7,22]. Previous investigations indicate that the thymus is lymphopoietic, the spleen is lymphopoietic thrombopoietic and erythropoietic [23,24], and the liver is granulopoietic with small lymphocyte-like cells in the perihepatic subcapsular region (PSR) which might be granulocyte precursors [7,23]. The newt liver possesses immunologic capabilities due to the presence of lymphocytes in the PSR of the liver [4].

This study has shown that the hematopoietic tissue structures of amphibian livers were observed in three regions: (a) the perihepatic subcapsular region (PSR), (b) portal triads region (PTR), and (c) inter-hepatic lobular nodule. Our study of 46 species showed that hematopoietic tissue structures were observed in both PSR and PTR in both Caudata and Gymnophiona orders, but in the order Anura, hematopoietic tissue was not observed in either PSR or PTR. Inter-hepatic lobular nodules were observed in all amphibian livers. In this study, we revealed that anuran livers did not have hematopoietic tissue structures, as did mammal liver. In contrast, urodele and caecilian livers had hematopoietic tissue structures with hepatic initial sites of hematopoiesis.

## Conclusions

This study showed that the architecture of the parenchymal arrangement was related to phylogenetic relationships, but hematopoiesis may not occur phylogenically. We suggested that hematopoietic tissue structures were concerned with the development in bone marrow and spleen of the systemic immune system. In hepatic ontogenesis, we demonstrated that the parenchymal arrangement is formed phylogenically.

## Competing interests

The authors declare that they have no competing interests.

## Authors’ contributions

HA and AI collected animals and made histological studies. HA conceived of the study, and participated in its design and draft the manuscript. AI carried out the histological staining and performed the morphometric analysis. All authors read and approved the final manuscript.
